# The Royal Hospital for Incurables

**Published:** 1921-05-14

**Authors:** James Carmichael

**Affiliations:** Redclyffe, Streatham Park, London, S.W. 16.


*To the Editor of* The Hospital.

### The Royal Hospital for Incurables.

*To the Editor of* The Hospital.

Sir,—May I ask you to be good and generous enough to give me an opportunity of putting the following before your readers? The high cost of maintenance, together with the unusually heavy claims on the beneficent work carried on by the Royal Hospital and Home for Incurables, Putney, has brought about a financial crisis. The Board of Management is faced with two alternatives—namely, it must either raise at least an additional £10,000 this year for maintenance, or curtail the splendid work it is now doing. The latter course would be nothing short of a national calamity.

My colleagues on the Board have urged me to take the chair at a dinner to be held on Thursday, June 9, in the Hall of the Grocers' Company, kindly lent for the occasion by the Master and Court.

This is not an ordinary appeal; it is forced upon us by dire necessity. We have now reached the limit with our bankers! The overdraft is a large one. Last year to help make ends meet we had to sell some of our very slender investments. That, of course, was only a temporary expedient, and cannot be repeated. The work must go on, and money must be found. The knowledge of an incurable illness is a heavy burden, but when combined with poverty it is surely a condition that merits our most generous support. Something must be done for the thousand patients now dependent on our efforts. I have sufficient faith in God and my fellow-men to feel sure that this appeal will not be made in vain, and I will gladly and promptly acknowledge any sum sent to me at Bond Court House, Walbrook, E.C. 4.—Your faithfully,

James Carmichael.

Redclyffe, Streatham Park, London, S.W. 16.

